# From blueprint to practice: an innovative dynamic 9-box grid improves exercise participation and aerobic capacity in graduate students

**DOI:** 10.3389/fpubh.2025.1672415

**Published:** 2025-11-04

**Authors:** Lei Chen, Aichun Li, Wenhao Chen, Hongzhuang Wang, Junlai Zhou, Mingyun Sun

**Affiliations:** ^1^School of Physical Education, Hainan Normal University, Haikou, Hainan, China; ^2^Haikou No. 1 Middle School, Haikou, Hainan, China; ^3^School of Physical Education, Anqing Normal University, Anqing, Anhui, China

**Keywords:** dynamic 9-box grid, graduate students, aerobic capacity, physical activity, feedback frequency, exercise self-efficacy, transtheoretical model

## Abstract

**Background:**

Graduate students face considerable health risks due to sedentary behavior and academic stress, often demonstrating a marked intention–behavior gap in physical activity (PA). This study developed a two-dimensional 9-box grid model based on total exercise volume (TEV) and aerobic capacity (AC) to compare the efficacy of weekly versus monthly feedback in improving AC and PA participation among graduate students.

**Methods:**

A quasi-experimental trial was conducted over eight weeks. Thirty-two graduate students, stratified by baseline AC evaluated in a 12-minute run test, were randomized into two arms, weekly feedback (*n* = 16) and monthly feedback (*n* = 16). Feedback was delivered through a dynamic 9-box grid that integrated weekly TEV on the *x*-axis and periodic AC on the y-axis. The primary outcomes were changes in the 12-minute run performance and TEV. Secondary outcomes included scores from the Exercise Identity Scale (EIS) and the Self-Efficacy for Exercise Scale (SEE-C). Thirty graduate students (*n* = 15 per group) completed the trial and were included in the final analysis.

**Results:**

Significant group × time interactions were observed for 12-min run distance (*F* = 4.29, *p* = 0.024, *η*^2^ = 0.241) and score (*F* = 6.49, *p* = 0.005, *η*^2^ = 0.325). The weekly feedback group demonstrated sustained improvements across all intervals (*p* < 0.001, Hedges’ *g* = 1.16–1.78), while the monthly group improved only post-intervention. Weekly feedback also resulted in significantly greater gains in self-efficacy (*p* = 0.044, *g* = 0.77) and higher TEV scores at multiple timepoints (*p* < 0.05). Both groups showed improved exercise identity (*p* < 0.001), with no between-group difference. Center of gravity analysis indicated greater migration toward healthier grid zones in the weekly group (ΔG = −1.93 vs. −1.47).

**Conclusion:**

The dynamic 9-box grid model effectively enhances aerobic capacity, promotes physical activity, and strengthens psychological outcomes through dual-axis evaluation and closed-loop feedback. Weekly feedback demonstrates superior efficacy in sustaining behavior modification and facilitating tier progression. This model provides a replicable, theory-informed strategy for health behavior management in graduate student populations.

## 1 Introduction

As the core cohort of knowledge workers, graduate students’ health capital accumulation directly impacts the sustainable development of academic productivity and innovative capacity (1). However, prevalent issues, including intensified research pressure and prolonged sedentary behavior, have led to marked increases in chronic diseases and psychological disorders among graduate students (2, 3). A national survey targeting Chinese graduate students revealed that only 21.2% maintained regular exercise participation, whereas 76.2% remained in “occasional participation” or “preparation stages” (4). This significant intention–behavior gap reflects deficiencies in behavioral maintenance mechanisms within conventional health intervention strategies (5, 6). Consequently, developing scientific intervention strategies to increase exercise motivation, optimize behavioral patterns, and improve physical fitness has become an urgent research priority.

Aerobic capacity (AC, also termed cardiopulmonary endurance or cardiorespiratory fitness) refers to the sustained ability to perform aerobic work, which integrally reflects oxygen uptake, transport, and utilization capacities. As a core component of physical fitness (7), AC serves as a crucial indicator of overall health status (8) and has been recognized as “the fifth clinical vital sign” (9). Empirical evidence indicates that prolonged sedentary behavior results in significantly lower AC levels in graduate students than those of age-matched population norms, triggering cascading effects, including impaired concentration and diminished academic efficiency (10). Current exercise interventions predominantly focus on prescription design but often lack systematic integration of theoretical constructs critical for behavioral maintenance phases, such as dynamic goal calibration and visual feedback mechanisms (11). This theoretical deficit has led to suboptimal exercise adherence and AC improvement outcomes in graduate student populations.

In recent years, performance management theory has shown unique potential in health behavior modification, facilitating behavioral change through goal setting, dynamic feedback mechanisms, and incentive systems (12, 13). Furthermore, the 9-box performance matrix has been widely used in talent management and leadership development (14). Developed by General Electric (GE) in the 1970s, this matrix categorizes employees into nine quadrants on the basis of their performance and potential, helping organizations identify key talents and develop targeted training programs. It evaluates talent through performance outcomes and developmental potential, providing a dual-axis framework for targeted developmental strategies. Empirical studies have shown that this model can significantly increase the proportion of high-performing talent, and its applicability has been verified in business management and healthcare services (15).

Crucially, the scientific value of the 9-box grid extends beyond classification functionality to its dynamic evolution mechanism. Through periodic assessments and data visualization dashboards, this system enables managers to map talent development trajectories, establishing a closed-loop management cycle of “evaluation-feedback-optimization.” This characteristic aligns with behavioral change theories in health management, where phased feedback enhances self-efficacy to drive the transition from “behavioral intention” to “action maintenance,” as per the Transtheoretical Model (16). However, existing research has focused predominantly on corporate contexts, with health promotion applications remaining largely theoretical (17). Critical gaps persist regarding dynamic multidimensional health competencies and comparative studies on feedback frequency optimization. Evidence-based research targeting special populations is particularly scarce, resulting in intervention strategies with insufficient alignment to target groups’ cognitive profiles (18).

Therefore, this study aims to develop and preliminarily validate a dynamic 9-box grid health management model to address the persistent intention-behavior gap and insufficient sustainability of feedback mechanisms among graduate students. This interdisciplinary framework incorporates wearable-monitored behavioral data (total exercise volume) and periodic aerobic capacity assessments via standardized running tests, forming a dual-axis evaluation system that enables personalized and tiered interventions. A central goal is to empirically compare the relative efficacy of weekly versus monthly feedback in enhancing exercise adherence, self-efficacy, and aerobic fitness. Ultimately, this research seeks to advance health promotion in higher education by innovatively adapting a corporate performance management tool for health behavior guidance.

## 2 Materials and methods

This study was conducted in accordance with the Declaration of Helsinki and was approved by the Ethics Committee of Anqing Normal University (Approval Number: AQNU2024110). All procedures, including participant recruitment and data collection, were carried out at Hainan Normal University with institutional support and collaboration. Prior to participation, all individuals provided written informed consent after being thoroughly informed of the study’s purpose, procedures, potential risks, and benefits. Participants were assured of their voluntary involvement, the right to withdraw at any time without penalty, and the confidentiality of their anonymized data.

### 2.1 Participants

This preliminary quasi-experimental study examined a novel dynamic 9-box grid management system. *A priori* power analysis (G*Power 3.1) indicated a minimum sample size of 27 participants (*α* = 0.05, 1−*β* = 0.80), based on previously reported standard deviations for aerobic capacity (19). Accounting for a 20% attrition rate, the target sample was set to 32.

**Figure 1:**
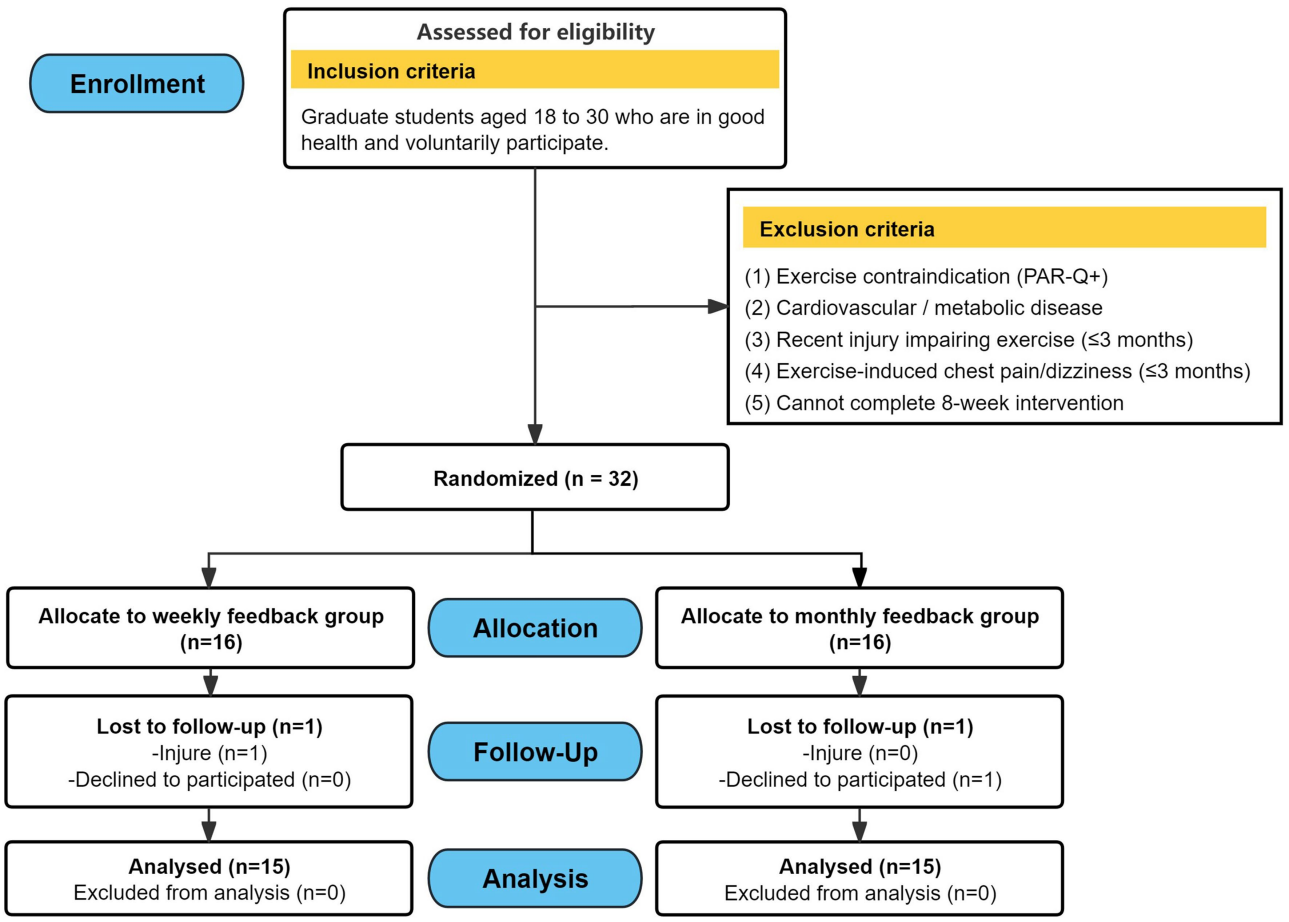
Flowchart of study participation and follow-up.

Participants were recruited through convenience sampling from a natural class of postgraduate students at Hainan Normal University between September 12 and October 1, 2024, under the supervision of the principal investigator (Li). Eligible participants were healthy postgraduate students aged 18–30 years. Exclusion criteria comprised cardiovascular disease, any contraindications identified via the Physical Activity Readiness Questionnaire (PAR-Q) (20), lower-limb injuries within the past 3 months, or symptoms of chest pain. Following baseline testing, eligible participants were stratified according to their 12-min running performance and randomly assigned to either a weekly feedback group (*n* = 16) or a monthly feedback group (*n* = 16). Personnel responsible for outcome assessment and statistical analysis were blinded to group assignment. The screening and allocation process is illustrated in Figure 1.

### 2.2 Experimental design

**Figure 2:**
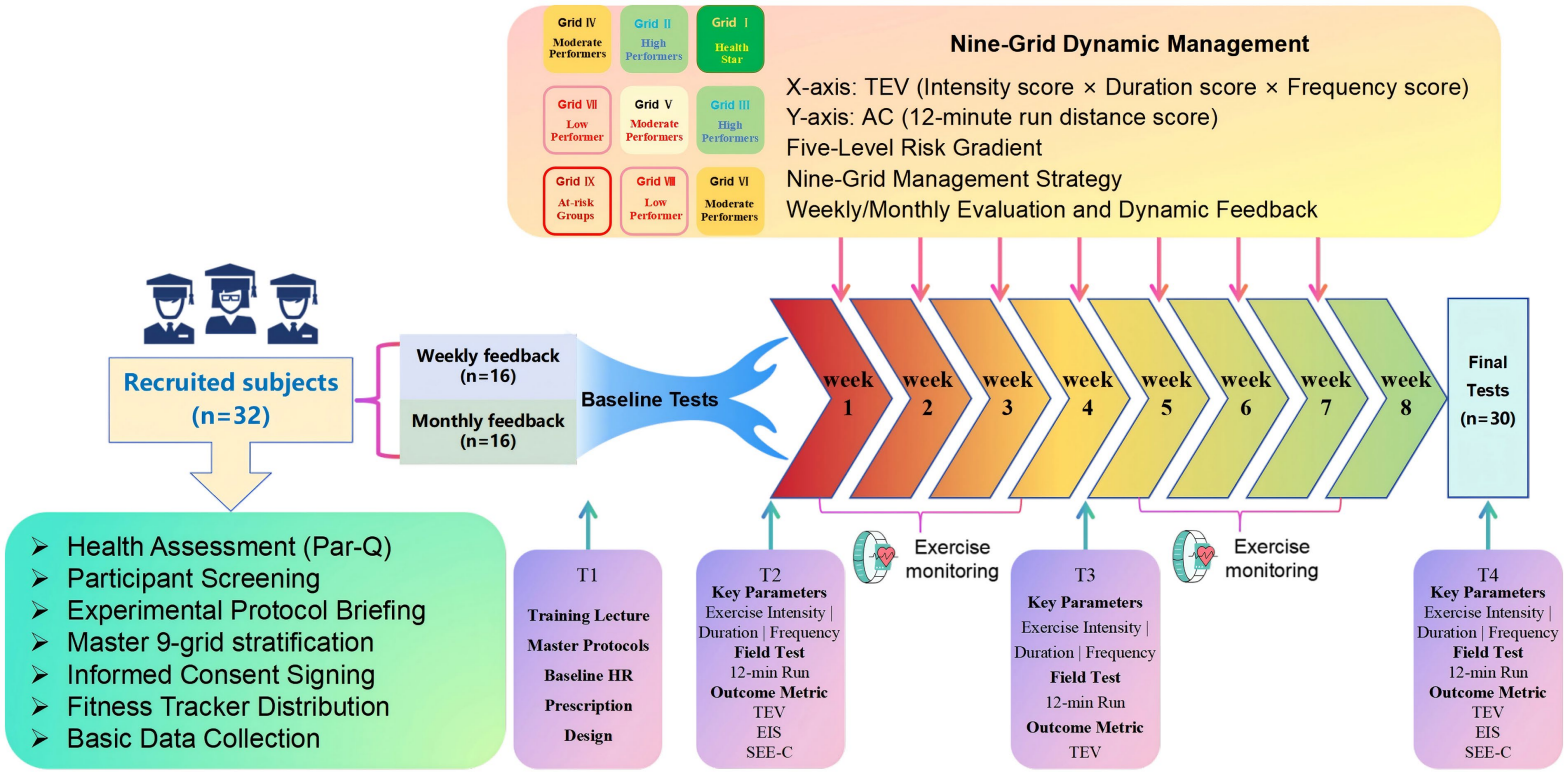
Experimental flow design.

A randomized, two-group, repeated-measures design was employed to evaluate the intervention. Aerobic capacity was assessed using the 12-min run test at baseline (week 0), mid-intervention (week 5), and post-intervention (week 9). Total exercise volume (TEV) was recorded weekly throughout the 8-week intervention. Exercise identity and self-efficacy were measured using validated scales at baseline and post-intervention. The overall experimental workflow is illustrated in Figure 2.

### 2.3 Methods of intervention

**Figure 3:**
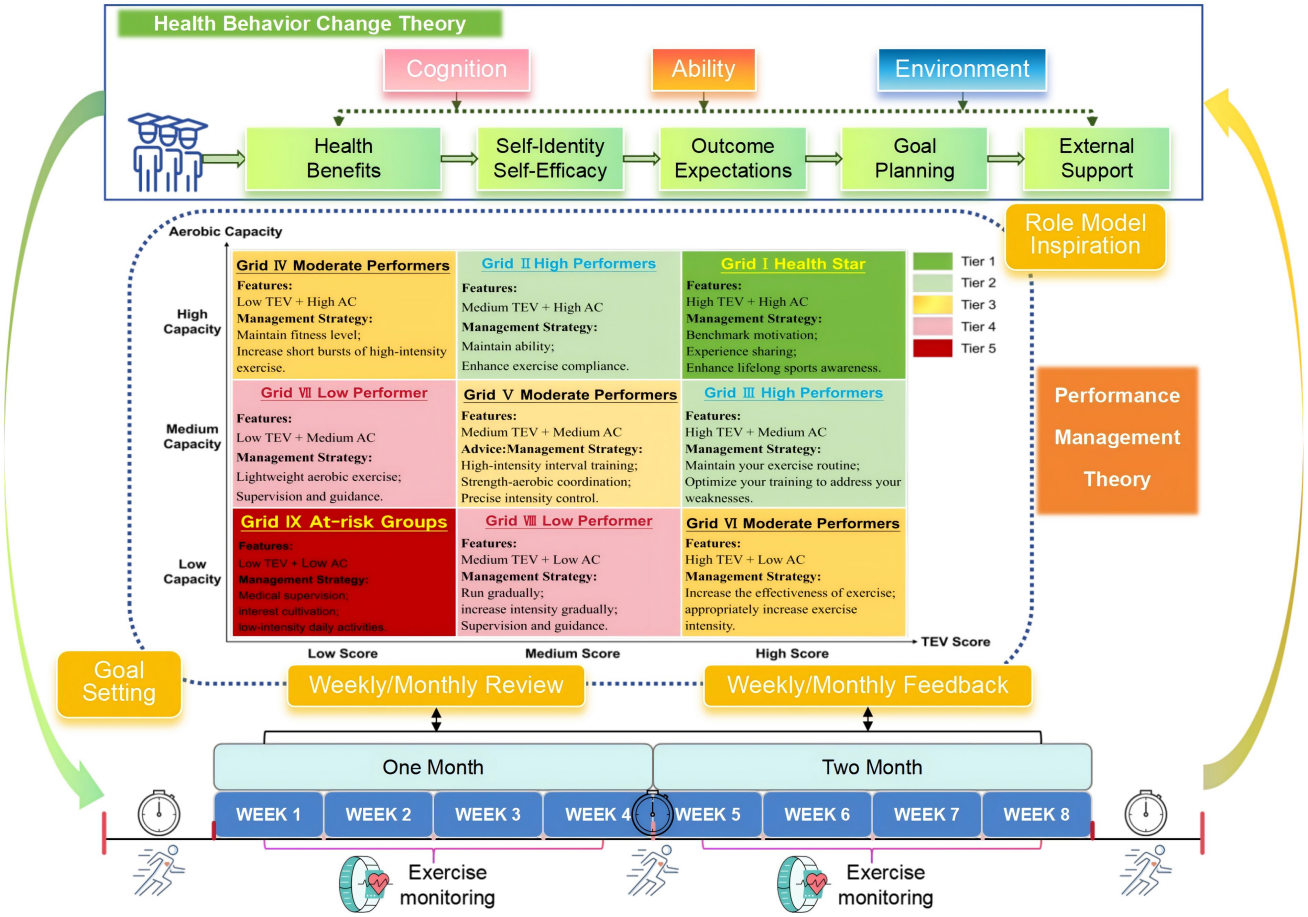
Aerobic capacity 9-grid management model integrating health behavior change and performance management theories.

Based on the integrated framework centered around the 9-box grid model (Figure 3), a comprehensive intervention was implemented. The stratification matrix was constructed according to established public health and exercise science standards, specifically based on ACSM guidelines (21) and Li’s aerobic capacity assessment framework (22). This model stratified participants by cross-referencing their TEV scores and 12-min run performance, both categorized into tertiles, which positioned each individual into one of nine distinct zones.

The management strategies for these zones were designed as a dynamic and tiered system aligned with health behavior change principles (23). Individuals identified as Health Star in Grid I with high motivation and high capacity received recognition and opportunities for experience sharing to bolster their self-identity and serve as benchmarks for others. Those in the High Performers grids (II & III) were provided with personalized goal planning to maintain their ability and address specific weaknesses, fostering continued growth through structured outcome expectations.

Participants in the intermediate grids (IV, V, VI) underwent optimized training regimens incorporating appropriate intensity progression and coordination exercises, supported by ongoing monitoring to stabilize exercise habits. For individuals in grids with lower motivation or capacity (VII & VIII), strategies focused on building self-efficacy through supervised guidance and lightweight activities. The most at-risk group in Grid IX received intensive one-on-one coaching involving psychological motivation and internal calibration to address initiative barriers.

To evaluate the effect of feedback timing within this model, all participants were educated on the grid rules and assessment criteria prior to the study. Each week, researchers dynamically updated participant grid positions based on the latest TEV scores. Consistent with the random assignment, the weekly feedback group received updated position and strategy recommendations each week, whereas the monthly feedback group received consolidated feedback only once at the beginning of each four-week cycle. This approach operationalized the closed-loop performance management process (24), integrating continuous assessment and tailored feedback to create a responsive intervention system aimed at sustaining behavior change.

### 2.4 Measurements

#### 2.4.1 Exercise volume measurement (*x*-axis)

Objective exercise data (intensity, duration, frequency) were collected via Huawei Band 6 (Model: FRA-B19), with raw datasets automatically exported and synchronized to a cloud-based intelligent management platform via the Huawei Health App. To ensure the reliability of the data, two trained researchers performed a single-blind check of exercise intensity, duration, and frequency on a predesigned exercise log sheet to eliminate recording bias through cross-validation. The validated data were transformed into weekly TEV scores every Monday morning via standardized algorithms, ensuring temporal validity and computational traceability through blockchain timestamping. The TEV metric was calculated as TEV = (intensity score) × (duration score) × (frequency score), where each dimension was normalized to a 0 ~ 100 scale via *z*-score transformation. TEV scores were stratified into tertiles: high (80 ~ 100), moderate (60 ~ 79), and low (60), with detailed scoring criteria per dimension provided in Supplementary Table S2.

#### 2.4.2 Aerobic capacity assessment (*y*-axis)

This study used a standardized 12-min run test to assess participants’ AC. Measurements were administered outdoors on a standard 400-m track. The participants wore the Huawei Band 6 to monitor exercise heart rate and intensity zones in real time, whereas the Keep application was employed for timing and dynamic tracking of running distance, enabling synchronized multidimensional data collection of exercise intensity, duration, and distance. All the data were cross-validated between the two systems to ensure an objective and accurate assessment of aerobic capacity. Performance was stratified into tertiles according to the Peking University Institute of Sports Science criteria: high (80 ~ 100), moderate (60 ~ 79), and low (60), with detailed classification parameters in Supplementary Table S3.

#### 2.4.3 Exercise identity evaluation

The Exercise Identity Scale (EIS), developed by Anderson and Cychosz was administered to evaluate exercise identity (25). This 9-item instrument employs a 7-point Likert scale (1 = “strongly disagree” to 7 = “strongly agree”), yielding summative scores from 9 to 63, with higher scores reflecting stronger exercise identity. The Cronbach’s alpha coefficient of the scale was 0.9, indicating excellent internal consistency reliability.

#### 2.4.4 Exercise self-efficacy measurement

The Chinese Self-Efficacy for Exercise Scale (SEE-C), translated by Lingling Li was used to measure the self-efficacy of the participants for exercise (26). The scale consists of 9 items, each of which is rated from 0 (not very confident) to 10 (very confident), and the total score ranges from 0 to 90 points. The scale has a Cronbach’s alpha coefficient of 0.75, demonstrating good internal consistency, and is suitable for assessing changes in subjects’ exercise self-efficacy in different contexts.

### 2.5 Data analysis

Statistical analyses were performed using SPSS 27 for data analysis, and GraphPad Prism 9 and Origin 2022 for graphing. Continuous data are presented as mean ± standard deviation (SD). Repeated-measures ANOVA with Greenhouse–Geisser correction for non-sphericity was applied to evaluate group × time interactions for primary outcomes, including 12-min run distance and score. Within-group changes were analyzed using paired *t*-tests, and between-group differences in secondary outcomes such as EIS and SEE-C scores were compared using independent *t*-tests. For significant interactions, Bonferroni *post-hoc* tests were conducted. Effect sizes are reported as partial eta-squared for ANOVA and Hedges’ *g* for *t*-tests, with a two-sided alpha level of 0.05 defining statistical significance. Furthermore, a weighted center of gravity index was computed based on tier numbering from 1 to 5 to assess spatial distribution shifts within the nine-square grid before and after the intervention.


$$G=\frac{\sum (\text{weight}\cdot \text{coordinate})\;}{\sum \text{weight}}=\frac{\sum (\mathrm{wi}\cdot \mathrm{xi})\;}{\sum \mathrm{wi}}$$

## 3 Results

### 3.1 Demographic characteristics

A total of 32 participants were initially recruited, but two withdrew (one from each group due to injury or voluntary withdrawal), leaving 30 participants included in the final analysis, comprising 22 males (73.3%) and 8 females (26.7%). The mean age was 23.63 ± 1.58 years, and the mean BMI was 22.49 ± 2.74 kg/m^2^. All participants were graduate students in humanities or social sciences, with no chronic diseases or exercise contraindications. Independent samples *t*-tests indicated no significant differences in baseline characteristics between the weekly and monthly reminder groups (Table 1).

**Table 1:** Demographic characteristics of the participants.

Characteristic	Total sample (*n* = 30)	Weekly feedback group (*n* = 15)	Monthly feedback group (*n* = 15)	*p*-value
Age (years)	23.63 ± 1.58	23.73 ± 1.79	23.53 ± 1.41	0.74
Sex, *n* (%)				1.00
Male	22 (73.3)	12 (75)	12 (75)	
Female	8 (26.7)	4 (25)	4 (25)	
BMI (kg/m^2^)	22.49 ± 2.74	21.67 ± 2.03	23.31 ± 3.15	0.10
Education level, *n* (%)				
Master’s student	30 (100)	15 (100)	15 (100)	
Field of study, *n* (%)				
Humanities/social sciences	30 (100)	15 (100)	15 (100)	
Chronic disease history, *n* (%)	0 (0)	0 (0)	0 (0)	
Exercise contraindications, *n* (%)	0 (0)	0 (0)	0 (0)	

### 3.2 Effects on 12-min run distance

Repeated-measures ANOVA revealed a significant main effect of time and a significant group × time interaction on 12-min run distance, but no main effect of group (Table 2). Analysis of simple effects demonstrated a clear differential pattern of improvement between the groups (Table 3). The weekly feedback group showed significant, large improvements between all consecutive timepoints (all *p* < 0.001, Hedges’ *g* ranging from 1.26 to 1.78). In contrast, the monthly feedback group significantly improved from baseline to post-test (*p* < 0.001, *g* = 1.43) and from mid-test to post-test (*p* < 0.001, *g* = 1.26), but not from baseline to mid-test (*p* = 0.059, *g* = 0.52).

**Table 2:** Repeated-measures ANOVA results for 12-min run distance and score between feedback groups.

Outcome	Time point	Weekly feedback group (*n* = 15)	Monthly feedback group (*n* = 15)	Time			Group			Time × group		
				*F*	*p*	*η^2^*	*F*	*p*	*η^2^*	*F*	*p*	*η^2^*
12-min run distance (m)	Baseline	2139.33 ± 270.22	2168.00 ± 226.15	41.45	<0.001^***^	0.754	0.64	0.429	0.022	4.29	0.024^*^	0.241
	Week 4	2369.07 ± 222.96	2246.93 ± 209.96									
	Week 8	2497.33 ± 189.49	2417.33 ± 160.20									
12-min run score	Baseline	42.67 ± 28.09	47.00 ± 24.19	37.64	<0.001^***^	0.736	0.47	0.50	0.016	6.49	0.005^**^	0.325
	Week 4	65.67 ± 19.07	52.33 ± 25.06									
	Week 8	77.00 ± 12.93	71.00 ± 18.54									

Data are presented as mean ± SD. A repeated-measures ANOVA was conducted to examine the effects of group, time, and group × time interaction. Greenhouse–Geisser correction was applied when sphericity was violated. ^*^p < 0.05, ^**^p < 0.01, ^***^p < 0.001.

**Table 3:** *Post hoc* pairwise comparisons of 12-min run performance between assessment time points.

Variable	Group	Comparison	Mean change	SD of difference	*t*-value	*p*-value	Hedges’ *g* (95% CI)
12-min run distance (m)	Weekly feedback	Baseline vs. mid-test	229.73	151.29	6.28	<0.001^***^	1.58 (0.81, 2.32)
		Baseline vs. post-test	358.00	249.78	7.10	<0.001^***^	1.78 (0.95, 2.59)
		Mid-test vs. post-test	128.27	182.99	5.03	<0.001^***^	1.26 (0.57, 1.93)
	Monthly feedback	Baseline vs. mid-test	78.93	148.66	2.06	0.059	0.52 (−0.02, 1.04)
		Baseline vs. post-test	249.33	170.02	5.68	<0.001^***^	1.43 (0.70, 2.13)
		Mid-test vs. post-test	170.40	131.75	5.01	<0.001^***^	1.26 (0.57, 1.92)
12-min run score	Weekly feedback	Baseline vs. mid-test	23.00	14.37	6.20	<0.001^***^	1.56 (0.79, 2.30)
		Baseline vs. post-test	34.33	20.95	6.35	<0.001^***^	1.60 (0.82, 2.35)
		Mid-test vs. post-test	11.33	9.54	4.60	<0.001^***^	1.16 (0.50, 1.79)
	Monthly feedback	Baseline vs. mid-test	5.33	14.33	1.44	0.171	0.36 (−0.15, 0.86)
		Baseline vs. post-test	24.00	15.38	6.05	<0.001^***^	1.52 (0.76, 2.25)
		Mid-test vs. post-test	18.67	14.57	4.96	<0.001^***^	1.25 (0.56, 1.91)

Data are presented as mean change ± SD of the difference. Simple effects were analyzed using paired-samples *t*-tests with Bonferroni correction. Effect sizes are reported as Hedges**’** *g* with 95% confidence intervals. ^***^p < 0.001.

### 3.3 Effects on 12-min run score

A similar pattern was observed for the 12-min run scores. Repeated-measures ANOVA indicated a significant main effect of time and a significant group × time interaction, with no main effect of group (Table 2). Simple effects analysis confirmed that the weekly feedback group significantly improved between all assessment points with large effects (all *p* < 0.001, Hedges’ *g* ranging from 1.16 to 1.60). The monthly feedback group, however, demonstrated significant score increases only from baseline to post-test (*p* < 0.001, *g* = 1.52) and from mid-test to post-test (*p* < 0.001, *g* = 1.25), with no significant change from baseline to mid-test (*p* = 0.171, *g* = 0.36). Detailed results are presented in Table 3.

### 3.4 Effects on exercise identity

**Figure 4:**
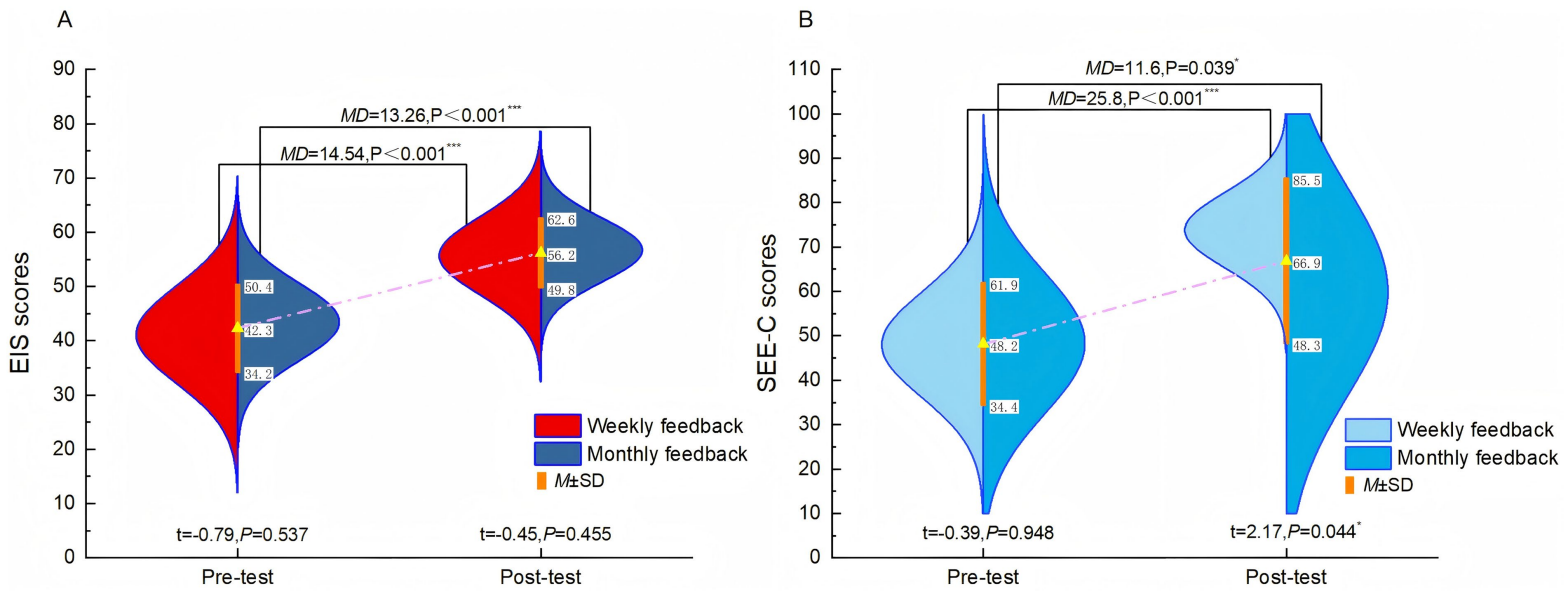
Effects of different feedback conditions on exercise identity and self-efficacy. **(A)**: Exercise Identity Scale (EIS) scores; **(B)**: Self-Efficacy for Exercise Scale (SEE-C) scores.

Both groups demonstrated significant, large improvements in EIS scores after the 9-grid management (both *p* < 0.001). No significant difference was found between the groups at post-test (*p* = 0.455), indicating that the feedback frequency did not have differing effects (see Table 4; Figure 4A).

**Table 4:** Comparison of EIS scores and SEE-C scores between two intervention groups before and after management.

Variable	Group	Pre-test	Post-test	Within-group comparison		Between-group comparison (post-test)	
				*t*-value, *p*-value	Hedges’ *g* (95% CI)	*t*-value, *p*-value	Hedges’ *g* (95% CI)
EIS score	Weekly feedback (*n* = 15)	41.13 ± 8.98	55.67 ± 7.12	*t* = 13.22, *p* < 0.001^***^	2.21 (4.66, 2.00)	*t* = −0.45, *p* = 0.455	−0.16 (−0.86, 0.54)
	Monthly feedback (*n* = 15)	43.47 ± 7.15	56.73 ± 5.86	*t* = 10.47, *p* < 0.001^***^	2.63 (3.70, 1.54)		
SEE-C score	Weekly feedback (*n* = 15)	48.00 ± 11.86	73.80 ± 8.49	*t* = 8.80, *p* < 0.001^***^	3.32 (3.15, 1.25)	*t* = 2.17, *p* = 0.044^*^	0.77 (0.39, 1.49)
	Monthly feedback (*n* = 15)	48.33 ± 15.85	59.93 ± 23.29	*t* = 2.28, *p* = 0.039^*^	0.57 (1.10, 0.03)		

Data are presented as mean ± SD. Within-group comparisons (pre vs. post) were analyzed using paired-samples *t*-tests. Between-group comparisons at post-test were analyzed using independent-samples *t*-tests. Effect sizes are reported as Hedges**’** *g* with 95% confidence intervals. *p* < 0.05, ***p < 0.001.

### 3.5 Effects on self-efficacy

Analysis of SEE-C scores revealed that while both groups improved after the 9-grid management, the weekly-feedback group exhibited a significantly greater increase than the monthly-feedback group at post-test (*p* = 0.044), with a large effect size (see Table 4; Figure 4B). This indicates that more frequent feedback is more effective in enhancing self-efficacy regarding exercise.

### 3.6 Temporal progression of TEV scores

**Figure 5:**
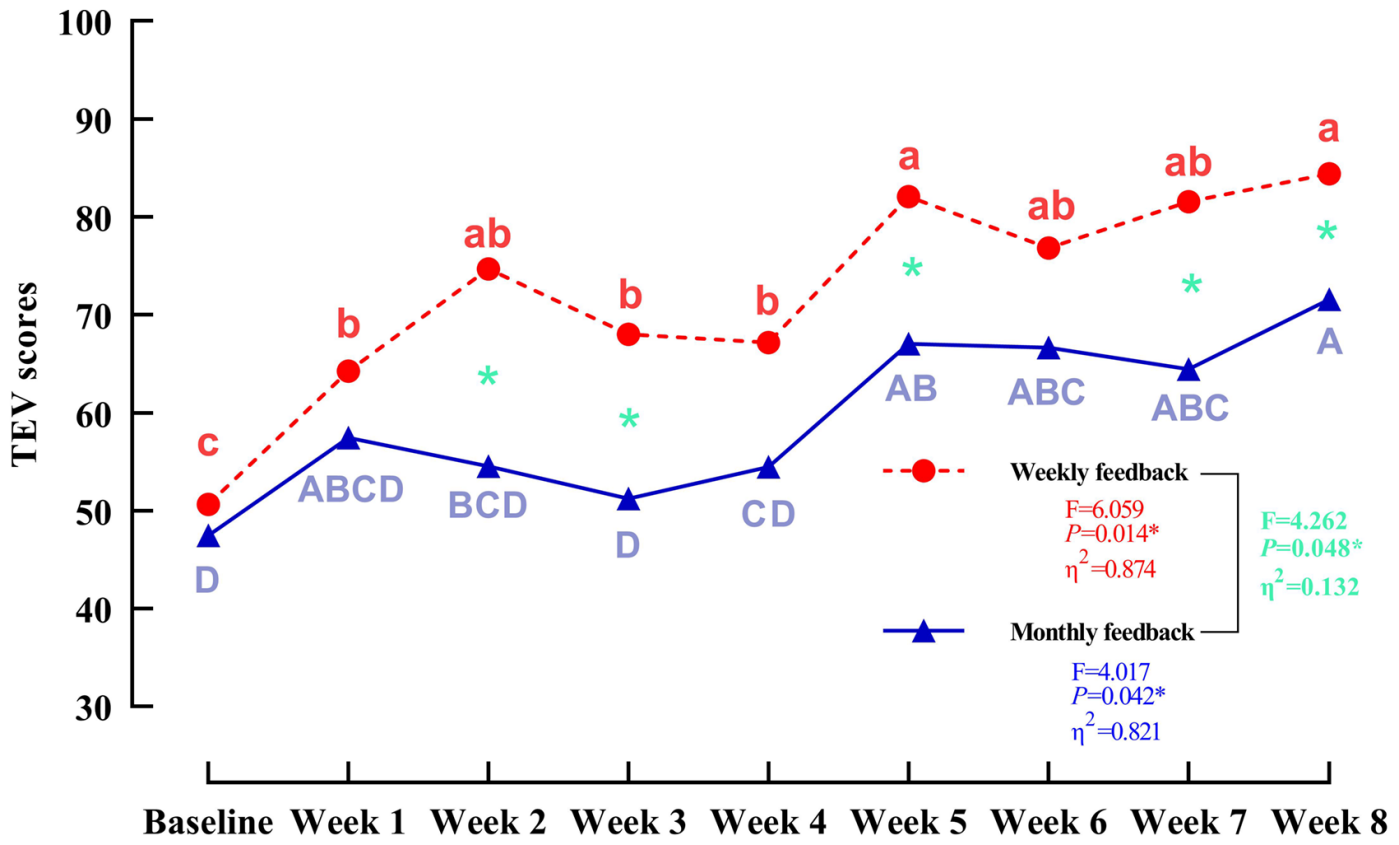
Temporal trajectories of the TEV scores under the 9-box grid intervention. “a, b, c” indicate different levels of significance within the weekly reminder group (*p* < 0.05); “A, B, C, D” indicate different levels of significance within the monthly reminder group (*p* < 0.05); green indicates a significant difference between the two groups (*p* < 0.05).

A repeated-measures ANOVA revealed a significant group × time interaction (*F* = 2.96, *p* = 0.022, *η*^2^ = 0.530) in TEV scores. As illustrated in Figure 5, while both groups showed an increasing trend over time, the weekly feedback group exhibited a more substantial improvement. *Post-hoc* between-group comparisons confirmed that the weekly group achieved significantly higher TEV scores than the monthly feedback group at multiple assessments (see Table 5), with medium to large effect sizes (Hedges’ *g* ranging from 0.90 to 0.97).

**Table 5:** Comparison of TEV scores between weekly and monthly feedback groups across multiple time points.

Time point	Weekly feedback group (*n* = 15)	Monthly feedback group (*n* = 15)	*t*-value	*p*-value	Hedges’ *g* (95% CI)
Baseline	50.67 ± 31.95	47.47 ± 34.17	0.27	0.793	0.09 (−0.60, 0.79)
Week 1	64.27 ± 29.63	57.47 ± 28.47	0.64	0.527	0.23 (−0.47, 0.93)
**Week 2**	**74.67 ± 17.67**	**54.53 ± 23.81**	**2.63**	**0.014** ^*^	**0.93 (0.19, 1.66)**
**Week 3**	**68.00 ± 16.77**	**51.20 ± 19.22**	**2.55**	**0.017** ^*^	**0.91 (0.16, 1.60)**
Week 4	67.20 ± 16.37	54.47 ± 19.23	1.95	0.061	0.69 (−0.31, 1.41)
**Week 5**	**82.07 ± 16.38**	**67.07 ± 17.77**	**2.61**	**0.014** ^*^	**0.93 (0.18, 1.66)**
Week 6	76.80 ± 14.36	66.67 ± 17.93	1.71	0.099	0.61 (−0.11, 1.32)
**Week 7**	**81.53 ± 15.43**	**64.47 ± 18.65**	**2.73**	**0.011** ^*^	**0.97 (0.22, 1.70)**
**Week 8**	**84.40 ± 13.27**	**71.60 ± 14.42**	**2.53**	**0.017** ^*^	**0.90 (0.16, 1.63)**
*F* value	6.059	4.017	–	–	–
*p*-value	*p* = 0.014^*^	*p* < 0.042^*^	–	–	–
*η* ^2^	0.874	0.821	–	–	–
Between-group	*F* = 4.262, *p* = 0.048^*^, *η*^2^ = 0.132				
Within-group	*F* = 9.658, *p* < 0.001^***^, *η*^2^ = 0.786				
Group × time	*F* = 2.959, *p* = 0.022^*^, *η*^2^ = 0.530				

Data are presented as mean ± SD. Between-group comparisons at each time point were analyzed using independent-samples *t*-tests. Longitudinal effects were examined by repeated-measures ANOVA (Greenhouse–Geisser corrected), with between-group, within-group, and interaction effects reported. Effect sizes are presented as Hedges’ *g* (for *t*-tests) with 95% CI or *η*^2^ (for ANOVA). *p < 0.05, ***p < 0.001. Bold values indicate a statistically significant difference between the two cue groups at the current time point.

### 3.7 Tier transition dynamics between promotion groups

**Figure 6:**
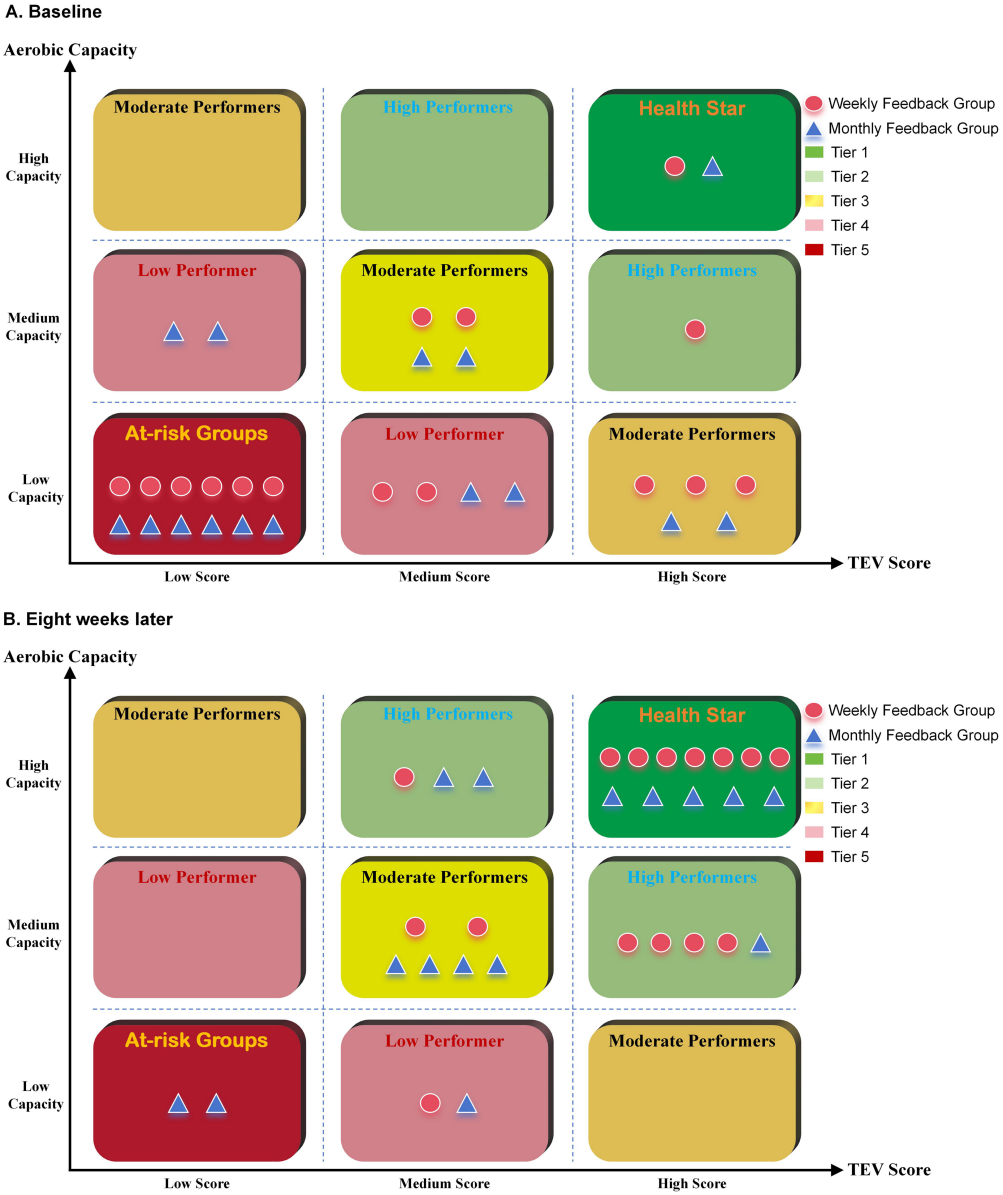
Hierarchical comparison of the two feedback groups before and after management. **(A)**: Before Management; **(B)**: After Eight Weeks of Implementation.

The changes in the center of gravity of the two feedback groups in the 9-box grid before and after management were compared to determine whether there was a trend of distribution shift (Table 6). The center of gravity of the weekly feedback group in the 9-box grid decreased from 3.73 before management to 1.80 after management, with a change of −1.93. The distribution of the number of prompts shifted significantly from the IV/V Tier to the top three tiers. The monthly feedback group’s center of gravity decreased from 3.93 to 2.47, with a change of −1.47, also showing a migration trend but with a smaller amplitude. These results indicate that the high-frequency feedback mechanism has a better promotional effect on optimizing tiered levels, particularly demonstrating outstanding efficacy in the conversion of low-performing talent. See Figure 6 for changes in trends before and after management. The complete baseline to eight-week management trend changes in the nine-grid ladder are shown in Supplementary Figures S1–S9.

**Table 6:** Comparison of changes in the center of gravity in the nine-square grid before and after management of the two feedback groups.

Group	Time point	Center of gravity	Change in center of gravity
Weekly feedback group	Baseline	3.73	−1.93
	Post-intervention	1.80	
Monthly feedback group	Baseline	3.93	−1.47
	Post-intervention	2.47	

## 4 Discussion

This study established a dual-dimensional 9-box grid management framework integrating exercise engagement and AC, examining the intervention effects of two feedback frequencies on graduate students’ AC and exercise behavior maintenance. The results demonstrated that the framework significantly enhanced AC and TEV, while concurrently reinforcing exercise identity and self-efficacy. Comparative analysis confirmed that weekly dynamic feedback produced superior beneficial effects on AC improvement and exercise habit formation relative to monthly feedback.

Regarding the mechanism of action, the 9-box grid management model facilitates precise regulation of AC adaptation through dynamic feedback. By integrating precise quantification of exercise volume (*X*-axis) and periodic assessment of AC (*Y*-axis), the model enables tailored adjustments of exercise plans and loads based on each subject’s position within the grid, thereby promoting more efficient stimulation of cardiopulmonary adaptive changes. Moreover, goal calibration based on matrix position changes addresses the static limitations of traditional exercise prescriptions (27). Previous studies have further confirmed that high-frequency feedback can improve the processing efficiency of the prefrontal cortex in responding to exercise reward signals and facilitate the adaptive remodeling of cardiopulmonary function (28). The more favorable progress trajectory observed in the weekly feedback group—as evidenced by greater improvements in the 12-min run distance and scores—further supports the practical utility of the dynamic 9-box grid feedback model in guiding participants to adjust training loads according to their grid position, thereby efficiently inducing cardiopulmonary adaptations.

In terms of exercise participation, the 9-box grid management model demonstrated unique effectiveness in promoting sustainable behavioral patterns. The steady growth trend in TEV and the significant shift in the hierarchical center of gravity index collectively indicate that this model effectively narrows the intention–behavior gap through dual psychological mechanisms. The significant improvement in EIS scores suggests that grid-based classification reinforces participants’ self-identification as “exercisers,” while the between-group differences in SEE-C scores underscore the critical role of feedback frequency. This finding aligns with the conclusion of Kuvaas et al. (29) that the perceived effectiveness of performance feedback is positively correlated with task performance—a correlation highly dependent on the timeliness and frequency of feedback. This principle is echoed in the medical field, where a lack of feedback has been shown to deter nurses from reporting errors, thereby hindering learning and improvement (30). In the present model, the weekly updates of grid positioning translate abstract health goals into concrete, hierarchical objectives. This allows participants to visually track their progress through changes in grid coordinates, thereby consistently activating intrinsic motivation. Furthermore, analysis of the temporal evolution of exercise persistence (TEV) revealed that the weekly feedback group established a stable upward trend after the fourth intervention week. This trend is consistent with the motivation–reward neural circuit theory proposed by Michaelsen and Esch (31), which posits that high-frequency positive reinforcement facilitates the formation of exercise habit circuits. This incentive mechanism, grounded in grid advancement, establishes a tight closed loop of “assessment–feedback–optimization,” continuously reinforcing participants’ successful experiences and bolstering their exercise confidence. Thus, it effectively translates performance commitment theory from corporate management into a sustainable driver for health behavior change.

Beyond the direct physical and behavioral outcomes, the observed improvements in exercise self-efficacy and identity suggest potential indirect benefits for mental well-being. The model’s structured feedback and achievement system may foster a sense of accomplishment and control—factors known to alleviate academic stress and enhance psychological resilience (32). Although mental health was not directly assessed in this study, the well-established link between physical activity and mental health suggests the potential of this model as a multifaceted intervention worthy of further investigation.

Compared with the traditional health management paradigm, the advantages of the 9-box grid management model lie in its efficiency in interdisciplinary integration. Through a systematic and modular structural design, it enables full-cycle and multidimensional intervention and support for managed subjects (33). Due to the lack of a dynamic calibration mechanism, exercise prescriptions in traditional models often lead to participant dropout because of suboptimal intervention effects or inadequate adaptability (34, 35). In contrast, the 9-box grid management model constructed in this study not only incorporates key components such as health assessment, behavioral intervention, and outcome feedback but also emphasizes management synergy, resource synergy, and information synergy, thereby significantly enhancing participant engagement and self-management capabilities. This dynamic adjustment mechanism compensates for the lagging response to changes in individual needs observed in traditional health management (36) and solves the bottleneck of difficulty in maintaining participation in long-term interventions caused by insufficient continuous feedback and flexible adaptation (37). Additionally, by virtue of multi-agent collaboration and data-driven decision support, the 9-box grid model not only improves the systematicness and sustainability of physical health management but also provides a scientifically grounded and efficient novel approach for the field of health management.

## 5 Limitations and future research directions

This study has several limitations. First, the sample size, though determined by a power analysis targeting a large effect, was relatively small and consisted primarily of male graduate students, which may somewhat limit the generalizability of the findings. The gender imbalance reflects the demographics of the source program; however, *post-hoc* analysis found no significant moderating effect of gender on the primary outcomes. Second, although the 8-week intervention duration was adequate for capturing initial changes, the lack of a no-intervention control group somewhat constrains the ability to establish definitive causal inferences. Nonetheless, the objective activity monitoring and the large effect sizes observed bolster the plausibility of an intervention-effect relationship. Finally, due to the lack of a comprehensive mental health assessment, it is not possible to determine whether the nine-grid management increases psychological stress.

Despite these limitations, this study offers encouraging initial evidence supporting the feasibility and efficacy of a dynamic 9-box grid model, originally adapted from corporate performance management, in promoting physical activity and fitness among graduate students. It emphasizes the importance of incorporating structured, feedback-driven frameworks into behavior change interventions and illustrates the promise of digital tools for personalized health management. Future research should feature randomized controlled trials with larger, more diverse populations, including a no-intervention control group. Such designs are crucial to firmly establish causality, further explore the differential effects of feedback timing, evaluate long-term sustainability, and include mental health assessments to thoroughly assess the intervention’s effectiveness.

## 6 Conclusion

This study innovatively developed a dynamic 9-box grid management model based on a two-dimensional assessment of total physical activity and aerobic capacity, and validated its effectiveness in graduate student health management through an 8-week intervention experiment. This 8-week proof-of-concept study demonstrates that the model effectively enhances aerobic capacity, exercise participation, and psychological resilience indicators through a closed-loop feedback mechanism, with weekly high-frequency feedback showing advantages in behavior maintenance. It provides a promising and replicable interdisciplinary practical framework for health management in higher education institutions. Future research should validate these findings in larger and more diverse populations over longer periods to establish long-term efficacy and explore integration with mental health support systems.

## Data Availability

The original contributions presented in the study are included in the article/Supplementary material, further inquiries can be directed to the corresponding authors.
