# Supplementary material for: From blueprint to practice: an innovative dynamic 9-box grid improves exercise participation and aerobic capacity in graduate students
Source: Front Public Health. 2025 Nov 4;13:1672415. doi: 10.3389/fpubh.2025.1672415 (PMC12623398; doi:10.3389/fpubh.2025.1672415)

Supplementary Material

CONTENT

1. Supplementary Table **......2**

Table S1 CONSORT 2010 checklist of information to include when reporting a randomised trial. .2

Table S2 Assessment dimensions and scoring criteria of the 9-box performance grid. 5

Table S3 Twelve-minute run evaluation criteria. 6

2. Supplementary Figure **...... 7**

Figure S1 Baseline tier distribution for the two prompt groups. 7

Figure S2 Tier distribution for the two prompt groups after 1 week of management. 7

Figure S3 Tier distribution for the two prompt groups after 2 weeks of management. 7

Figure S4 Tier distribution for the two prompt groups after 3 weeks of management. 8

Figure S5 Tier distribution for the two prompt groups after 4 weeks of management. 8

Figure S6 Hierarchical levels of the two prompt groups after 5 weeks of management. 8

Figure S7 Hierarchical levels of the two prompt groups after 6 weeks of management. 9

Figure S8 Hierarchical levels of the two prompt groups after 7 weeks of management. 9

Figure S9 Hierarchical levels of the two prompt groups after 8 weeks of management. 9

1. Supplementary Table

Table S1 CONSORT 2010 checklist of information to include when reporting a randomised trial.

CONSORT 2010 checklist of information to include when reporting a randomised trial*

Section/Topic	Item No	Checklist item	Reported on page No
**Title and abstract**			
	1a	Identification as a randomised trial in the title	1
	1b	Structured summary of trial design, methods, results, and conclusions (for specific guidance see CONSORT for abstracts)	1
Background and objectives	2a	Scientific background and explanation of rationale	1, 4-5
	2b	Specific objectives or hypotheses	1,5
Trial design	3a	Description of trial design (such as parallel, factorial) including allocation ratio	
	3b	Important changes to methods after trial commencement (such as eligibility criteria), with reasons	5-6
Participants	4a	Eligibility criteria for participants	Not applicable
	4b	Settings and locations where the data were collected	5
Interventions	5	The interventions for each group with sufficient details to allow replication, including how and when they were actually administered	5
Outcomes	6a	Completely defined pre-specified primary and secondary outcome measures, including how and when they were assessed	7-8
	6b	Any changes to trial outcomes after the trial commenced, with reasons	Not applicable
Sample size	7a	How sample size was determined	5

	7b	When applicable, explanation of any interim analyses and stopping guidelines	Not applicable
Randomisation:			
Sequence generation	8a	Method used to generate the random allocation sequence	5
	8b	Type of randomisation; details of any restriction (such as blocking and block size)	5
Allocation concealment mechanism	9	Mechanism used to implement the random allocation sequence (such as sequentially numbered containers), describing any steps taken to conceal the sequence until interventions were assigned	5
Implementation	10	Who generated the random allocation sequence, who enrolled participants, and who assigned participants to interventions	5
Blinding	11a	If done, who was blinded after assignment to interventions (for example, participants, care providers, those assessing outcomes) and how4	5
	11b	If relevant, description of the similarity of interventions	6-7
Statistical methods	12a	Statistical methods used to compare groups for primary and secondary outcomes	8
	12b	Methods for additional analyses, such as subgroup analyses and adjusted analyses	8
Participant flow (a diagram is strongly recommended)	13a	For each group, the numbers of participants who were randomly assigned, received intended treatment, and were analysed for the primary outcome	5, 11
	13b	For each group, losses and exclusions after randomisation, together with reasons	5, 11
Recruitment	14a	Dates defining the periods of recruitment and follow-up	5
	14b	Why the trial ended or was stopped	Not applicable
Baseline data	15	A table showing baseline demographic and clinical characteristics for each group	11
Numbers analysed	16	For each group, number of participants (denominator) included in each analysis and whether the analysis was by original assigned groups	11
Outcomes and estimation	17a	For each primary and secondary outcome, results for each group, and the estimated effect size and its precision (such as 95% confidence interval)	11-12
	17b	For binary outcomes, presentation of both absolute and relative effect sizes is recommended	Not applicable
Ancillary analyses	18	Results of any other analyses performed, including subgroup analyses and adjusted analyses, distinguishing pre-specified from exploratory	12
Harms	19	All important harms or unintended effects in each group (for specific guidance see CONSORT for harms)	Not applicable

Limitations	20	Trial limitations, addressing sources of potential bias, imprecision, and, if relevant, multiplicity of analyses	14
Generalisability	21	Generalisability (external validity, applicability) of the trial findings	14-15
Interpretation	22	Interpretation consistent with results, balancing benefits and harms, and considering other relevant evidence	13-14
**Other information**			
Registration	23	Registration number and name of trial registry	15
Protocol	24	Where the full trial protocol can be accessed, if available	ClinicalTrials.gov
Funding	25	Sources of funding and other support (such as supply of drugs), role of funders	15

Citation: Schulz KF, Altman DG, Moher D, for the CONSORT Group. CONSORT 2010 Statement: updated guidelines for reporting parallel group randomised trials. BMC Medicine. 2010;8:18. © 2010 Schulz et al. This is an Open Access article distributed under the terms of the Creative Commons Attribution License (http://creativecommons.org/licenses/by/2.0), which permits unrestricted use, distribution, and reproduction in any medium, provided the original work is properly cited.

*We strongly recommend reading this statement in conjunction with the CONSORT 2010 Explanation and Elaboration for important clarifications on all the items. If relevant, we also recommend reading CONSORT extensions for cluster randomised trials, non-inferiority and equivalence trials, non-pharmacological treatments, herbal interventions, and pragmatic trials. Additional extensions are forthcoming: for those and for up-to-date references relevant to this checklist, see www.consort-statement.org.

Table S2 Assessment dimensions and scoring criteria of the 9-box performance grid.

Indicator	Category	Score
Exercise Intensity	Vigorous (60 ~ <90% HRR)	5
	Moderate + Vigorous Intensity	4
	Moderate (40 ~ <60% HRR)	3
	Light (30 ~ <40% HRR)	2
	Very Light (<30% HRR)	1
Duration	Daily ≥30 min or Weekly ≥150 min	4
	Daily 20 ~ <30 min or Weekly ≥75 min	3
	Daily 10 ~ <20 min	2
	Daily <10 min	1
Frequency	>5 sessions/week	5
	3 ~ 5 sessions/week	4
	1 ~ 2 sessions/week	3
	2 ~ 3 sessions/month	2
	1 session/month	1
Criteria for evaluating exercise effectiveness and exercise performance		
TEV Score	Evaluation	Exercise Performance
80 ~ 100 points	Excellent engagement & scientific basis	High
60 ~ <80 points	Moderate engagement & scientific basis	Moderate
<60 points	Poor engagement & scientific basis	Low
**TEV = (Intensity score) × (Duration score) × (Frequency score)**		

Adapted from Li et al. (2023) (1).

Table S3 Twelve-minute run evaluation criteria.

Grade	Male (m)	Score	Female (m)
Excellent ~ Good	≥ 2800	100	≥ 2400
	2750 ~ < 2800	95	2350 ~ < 2400
	2700 ~ < 2750	90	2300 ~ < 2350
	2650 ~ < 2700	85	2250 ~ < 2300
	**2600 ~ < 2650**	**80**	**2200 ~ < 2250**
Pass	2550 ~ < 2600	75	2150 ~ < 2200
	2500 ~ < 2550	70	2100 ~ < 2150
	2450 ~ < 2500	65	2050 ~ < 2100
	**2400 ~ < 2450**	**60**	**2000 ~ < 2050**
Fail	2350 ~ < 2400	55	1950 ~ < 2000
	2300 ~ < 2350	50	1900 ~ < 1950
	2250 ~ < 2300	45	1850 ~ < 1900
	2200 ~ < 2250	40	1800 ~ < 1850
	2150 ~ < 2200	35	1750 ~ < 1800
	2100 ~ < 2150	30	1700 ~ < 1750
	< 2100	10	< 1700

Adapted from the Physical Education Teaching and Research Department of Peking University (2); Scoring system based on 100-point scale.

Reference:

1.Wang Z. ACSM Guidelines for Exercise Testing and Exercise Prescription: Beijing Sports University Press; 2019.

2.Li A, Zhou Y, Zhou J, Tian H, Wang H, Li X. The issue examination and innovation construction of evaluation index system on aerobic capacity for Chinese undergraduates under the perspective of validity. Journal of Physical Education. (2023);306. doi: 10.16237/j.cnki.cn44-1404/g8.2023.06.009.

2. *Supplementary Figure*

Figure S1 Baseline tier distribution for the two prompt groups.

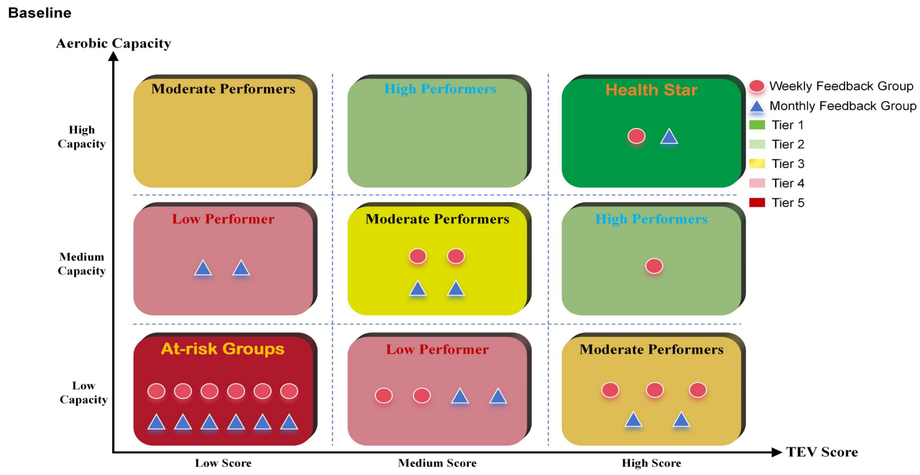

Figure S2 Tier distribution for the two prompt groups after 1 week of management.

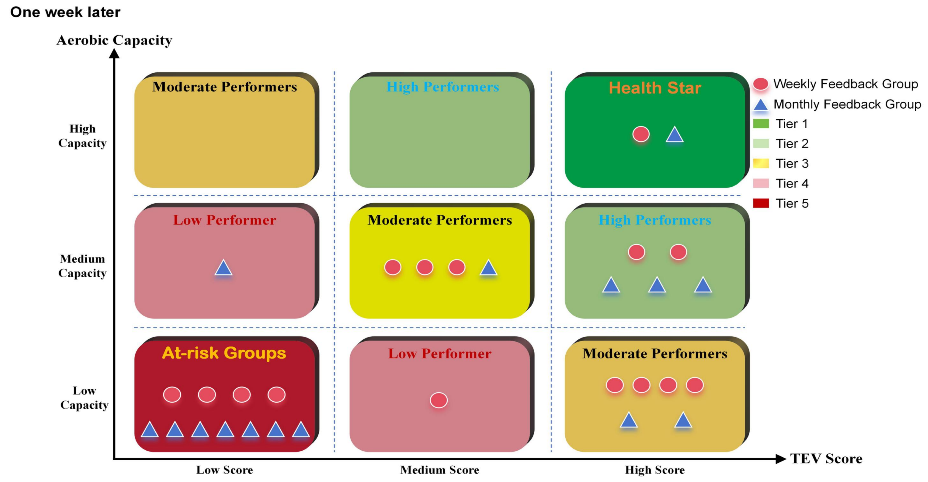

Figure S3 Tier distribution for the two prompt groups after 2 weeks of management.

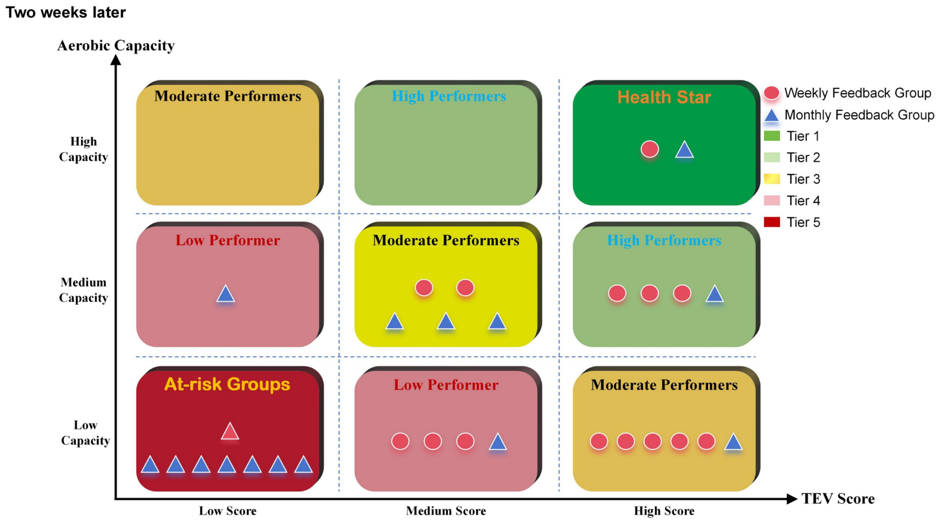

Figure S4 Tier distribution for the two prompt groups after 3 weeks of management.

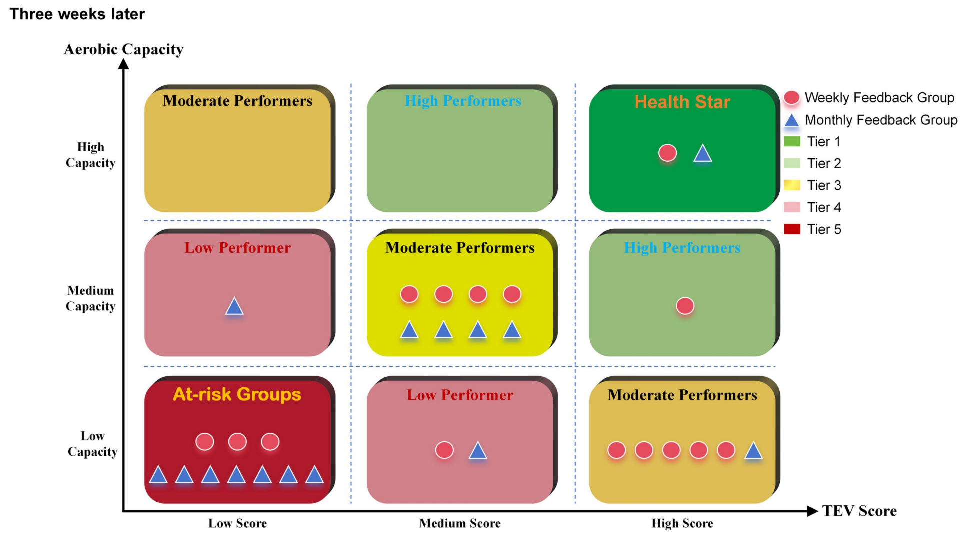

Figure S5 Tier distribution for the two prompt groups after 4 weeks of management.

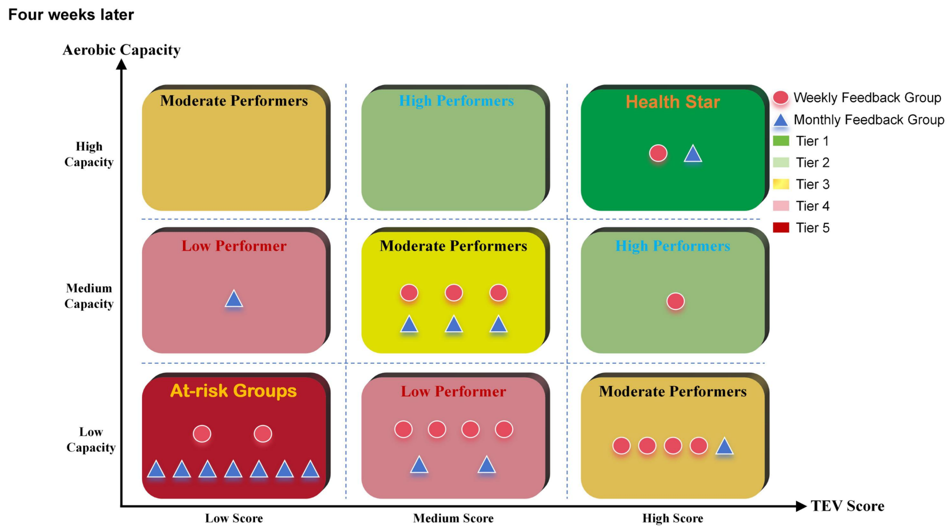

Figure S6 Hierarchical levels of the two prompt groups after 5 weeks of management.

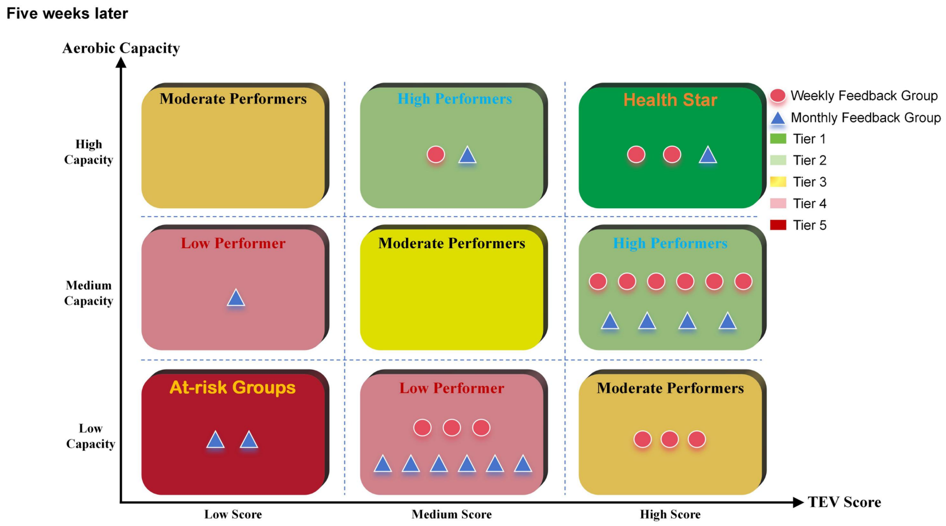

Figure S7 Hierarchical levels of the two prompt groups after 6 weeks of management.

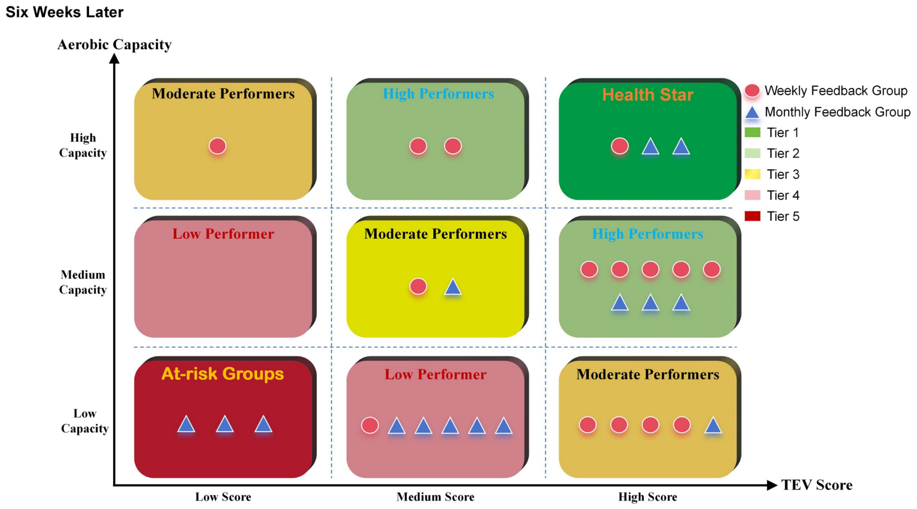

Figure S8 Hierarchical levels of the two prompt groups after 7 weeks of management.

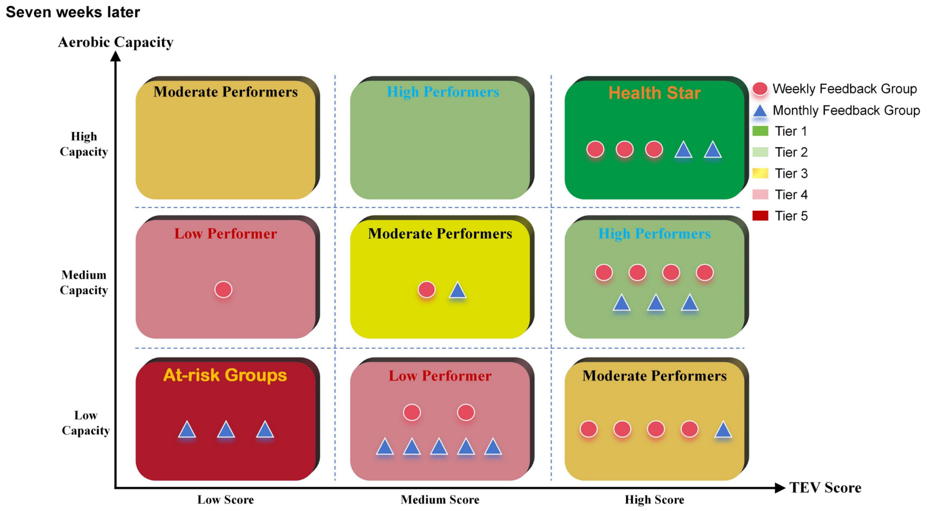

Figure S9 Hierarchical levels of the two prompt groups after 8 weeks of management.

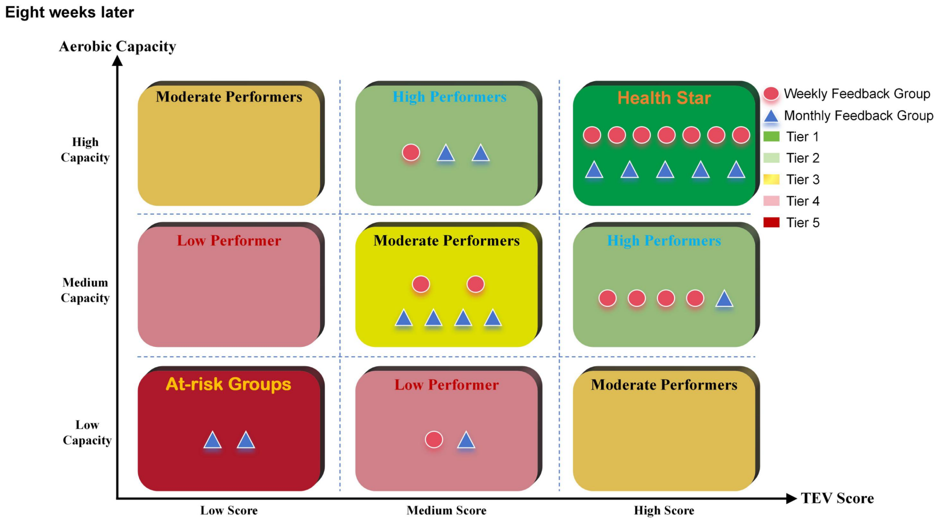

Supplement: Supplementary file 1 [file Data_Sheet_1.pdf]
